# Left Ventricular Unloading Is Associated With Lower Mortality in Patients With Cardiogenic Shock Treated With Venoarterial Extracorporeal Membrane Oxygenation: Results From an International, Multicenter Cohort Study

**DOI:** 10.1161/CIRCULATIONAHA.120.048792

**Published:** 2020-10-09

**Authors:** Benedikt Schrage, Peter Moritz Becher, Alexander Bernhardt, Hiram Bezerra, Stefan Blankenberg, Stefan Brunner, Pascal Colson, Gaston Cudemus Deseda, Salim Dabboura, Dennis Eckner, Matthias Eden, Ingo Eitel, Derk Frank, Norbert Frey, Masaki Funamoto, Alina Goßling, Tobias Graf, Christian Hagl, Paulus Kirchhof, Danny Kupka, Ulf Landmesser, Jerry Lipinski, Mathew Lopes, Nicolas Majunke, Octavian Maniuc, Daniel McGrath, Sven Möbius-Winkler, David A. Morrow, Marc Mourad, Curt Noel, Peter Nordbeck, Martin Orban, Federico Pappalardo, Sandeep M. Patel, Matthias Pauschinger, Vittorio Pazzanese, Hermann Reichenspurner, Marcus Sandri, P. Christian Schulze, Robert H.G. Schwinger, Jan-Malte Sinning, Adem Aksoy, Carsten Skurk, Lukasz Szczanowicz, Holger Thiele, Franziska Tietz, Anubodh Varshney, Lukas Wechsler, Dirk Westermann

**Affiliations:** 1 Departments of Cardiology (B.S., P.M.B., S. Blankenberg, S.D., A.G., P.K., D.W.), University Heart and Vascular Center Hamburg, Germany.; 2 Cardiothoracic Surgery (A.B., H.R.), University Heart and Vascular Center Hamburg, Germany.; 3 German Center for Cardiovascular Research (DZHK), partner site Hamburg/Lübeck/Kiel, Germany (B.S., P.M.B., A.B., S. Blankenberg, S.D., M.E., I.E., D.F., N.F., T.G., P.K., C.N., D.W.).; 4 Tampa General Hospital, University of South Florida (H.B.).; 5 Medizinische Klinik und Poliklinik I (S. Brunner, D.K., M.O.), LMU Klinikum, Munich, Germany.; 6 Herzchirurgische Klinik und Poliklinik (C.H.), LMU Klinikum, Munich, Germany.; 7 Department of Anesthesiology and Critical Care Medicine, CHU Montpellier, University Montpellier, France (P.C., M.M.).; 8 Division of Anesthesia, Critical Care and Pain Medicine (G.C.D.), Massachusetts General Hospital, Boston.; 9 Division of Cardiac Surgery (M.F., D.M.), Massachusetts General Hospital, Boston.; 10 Department of Cardiology, Paracelsus Medical University Nürnberg, Germany (D.E., M.P.).; 11 Department of Internal Medicine III, Cardiology and Angiology, University Hospital Schleswig-Holstein, Kiel, Germany(M.E., D.F., N.F., C.N.).; 12 University Heart Center Lübeck, University Hospital Schleswig-Holstein, Germany (I.E., T.G.).; 13 Institute of Cardiovascular Sciences, University of Birmingham and University Hospitals Birmingham and Sandwell and West Birmingham National Health ServiceTrusts, United Kingdom (P.K.).; 14 Department of Cardiology, Campus Benjamin, Charité Universitätsmedizin Berlin, Germany (U.L., C.S.).; 15 Franklin/German Centre for Cardiovascular Research (DZHK), partner site Berlin/Institute of Health (BIH), Germany (U.L., C.S.).; 16 Department of Internal Medicine, University of California, San Diego (J.L.).; 17 Cardiovascular Division, Department of Medicine, Brigham and Women’s Hospital, Harvard Medical School, Boston, MA (M.L., D.A.M., A.V.).; 18 Department of Internal Medicine and Cardiology, Heart Center Leipzig at University of Leipzig and Leipzig Heart Institute, Germany (N.M., M.S., L.S., H.T., F.T.).; 19 Department of Internal Medicine I, University Hospital Würzburg, Germany (O.M., P.N.).; 20 Department of Internal Medicine I, University Hospital Jena, Germany (S.M.-W., P.C.S.).; 21 Advanced Heart Failure and Mechanical Circulatory Support Program, Vita Salute University, Milan, Italy (F.P., V.P.).; 22 Department of Anesthesia and Intensive Care, IRCCS (Istituto di Ricovero e Cura a Carattere Scientifico) ISMETT (Istituto Mediterraneo trapianti e terapie avanzate), UPMC (University of Pittsburgh Medical Center)Italy, Palermo, Italy (F.P.).; 23 Department of Interventional Cardiology, St. Rita’s Medical Center, Lima, OH (S.M.P.).; 24 Medizinische Klinik II, Klinikum Weiden, Germany (R.H.G.S., L.W.).; 25 University Heart Center Bonn, Department of Cardiology, Germany (J.-M.S., A.A.).

**Keywords:** extracorporeal membrane oxygenation, shock, cardiogenic

## Abstract

**Background:**

Venoarterial extracorporeal membrane oxygenation (VA-ECMO) is increasingly used to treat cardiogenic shock. However, VA-ECMO might hamper myocardial recovery. The Impella unloads the left ventricle. This study aimed to evaluate whether left ventricular unloading in patients with cardiogenic shock treated with VA-ECMO was associated with lower mortality.

**Methods:**

Data from 686 consecutive patients with cardiogenic shock treated with VA-ECMO with or without left ventricular unloading using an Impella at 16 tertiary care centers in 4 countries were collected. The association between left ventricular unloading and 30-day mortality was assessed by Cox regression models in a 1:1 propensity score–matched cohort.

**Results:**

Left ventricular unloading was used in 337 of the 686 patients (49%). After matching, 255 patients with left ventricular unloading were compared with 255 patients without left ventricular unloading. In the matched cohort, left ventricular unloading was associated with lower 30-day mortality (hazard ratio, 0.79 [95% CI, 0.63–0.98]; *P*=0.03) without differences in various subgroups. Complications occurred more frequently in patients with left ventricular unloading: severe bleeding in 98 (38.4%) versus 45 (17.9%), access site–related ischemia in 55 (21.6%) versus 31 (12.3%), abdominal compartment in 23 (9.4%) versus 9 (3.7%), and renal replacement therapy in 148 (58.5%) versus 99 (39.1%).

**Conclusions:**

In this international, multicenter cohort study, left ventricular unloading was associated with lower mortality in patients with cardiogenic shock treated with VA-ECMO, despite higher complication rates. These findings support use of left ventricular unloading in patients with cardiogenic shock treated with VA-ECMO and call for further validation, ideally in a randomized, controlled trial.

## Abstract 2

Supplemental Digital Content is available in the text.

**Clinical Perspective**

**What Is New?**

- In this international, multicenter cohort study of 686 patients with cardiogenic shock treated with venoarterial extracorporeal membrane oxygenation, use of left ventricular unloading was associated with lower mortality.
- Use of left ventricular unloading in these patients was also associated with higher risk of complications, such as severe bleeding or interventions because of access site–related ischemia.

**What Are the Clinical Implications?**

- Our study supports use of left ventricular unloading in patients with cardiogenic shock treated with venoarterial extracorporeal membrane oxygenation.
- These findings indicate the need for evaluation of this treatment strategy in a randomized, controlled trial.

**Editorial, see p 2107**

Cardiogenic shock is associated with a high mortality rate of up to 50%.^1,2^ Over the past decades, venoarterial extracorporeal membrane oxygenation (VA-ECMO) has been increasingly used to treat cardiogenic shock,^2,3^ but the effect of VA-ECMO on mortality is unclear.^2,4^ VA-ECMO increases left ventricular (LV) pressure attributable to retrograde aortic perfusion. This could slow myocardial recovery or damage the myocardium and negatively affect survival.^5^

LV unloading using percutaneous assist devices has been suggested as an approach to address the increased afterload present in patients supported by VA-ECMO.^6^ By decreasing LV pressure (eg, unloading the LV), these assist devices might facilitate myocardial recovery, increase the probability of successful VA-ECMO weaning, and could ultimately lead to improved survival in cardiogenic shock.^6^

The Impella microaxial flow pump (Abiomed, Danvers, MA) is a catheter-based LV assist device that is inserted into the LV cavity by arterial access (predominantly femoral access in cardiogenic shock). From that position, it actively drains blood from the LV and propels it into the proximal ascending aorta, thereby decreasing LV preload and increasing cardiac output.^5^ It has been suggested that addition of an Impella to VA-ECMO (ECMELLA) might be feasible to treat patients with cardiogenic shock and could improve outcome.^7,8^ Although this approach holds promise on the basis of pathophysiology, the published evidence comparing VA-ECMO alone to an ECMELLA strategy is limited to case reports and smaller studies.^6,9,10^

The primary aim of this study was to evaluate outcome in an international, multicenter registry of patients with cardiogenic shock treated with VA-ECMO with or without LV unloading using an Impella. Secondary aims were to examine prespecified subgroups as well as the overall complication rate with both approaches.

## Methods

The data that support the findings of this study are available from the corresponding author on reasonable request.

### Setting

Consecutive patients with cardiogenic shock treated with either VA-ECMO alone or ECMELLA between 2005 and 2019 from 16 centers in 4 countries were retrospectively enrolled (STOP-SHOCK [Safety and Outcome of Contemporary Treatment Strategies for Cardiogenic Shock]; URL:https://www.clinicaltrials.gov. Unique identifier: NCT03313687). All participating hospitals are large centers experienced in the treatment of cardiogenic shock in general and in the use of mechanical circulatory support devices in particular. Cardiogenic shock was defined at the discretion of the local investigator. Cardiogenic shock after cardiotomy and age <18 years were the only exclusion criteria for this registry. Patients were treated at the discretion of the local investigators and per local guidelines. Baseline was defined as implantation of first device and variables were recorded in a dedicated database.

The study was conducted in accordance with the Declaration of Helsinki and was approved by local ethics committees and institutional review committees. The need for informed consent was waived by the main ethics committee because this was a retrospective analysis and only completely anonymized data were collected and analyzed.

This study was designed by the authors, who also gathered and analyzed the data, vouch for this study, wrote the article, and ultimately decided to publish. No company was involved in any part of this process.

### End Points

The primary end point of this study was 30-day all-cause mortality (events that occurred after 30 days were censored).

Safety end points were chosen to assess bleeding complications (severe/moderate bleeding defined by GUSTO [Global Utilization of Streptokinase and Tissue Plasminogen Activator for Occluded Coronary Arteries] criteria; intracerebral bleeding or hemorrhagic stroke on computed tomography; intervention because of bleeding; hemolysis, defined as lactate dehydrogenase ≥1000 U/L and haptoglobin <0.3 g/L in 2 consecutive samples within 24 hours), ischemic complications (ischemic stroke on computed tomography; intervention because of access site–related ischemia; laparotomy because of abdominal compartment or bowel ischemia), and other complications (hypoxic brain damage on computed tomography; renal replacement therapy; sepsis, defined as systemic inflammatory response syndrome criteria and ≥2 positive blood cultures).

### Statistical Analyses

Missing data were handled by multiple imputations with chained equations (R-package mice; 10 imputed data sets; variables used for the multiple imputation are shown in Table 1).^11^ In the imputed data sets, propensity scores for ECMELLA were calculated by a logistic regression model and then averaged. The following variables were used for the calculation of the propensity score: age (categorized), sex, cause of cardiogenic shock, previous cardiac arrest, VA-ECMO–assisted cardiopulmonary resuscitation (eCPR), mean blood pressure (categorized), heart rate (categorized), lactate (categorized), and pH (categorized). On the basis of these propensity scores, patients treated with ECMELLA were matched 1:1 to patients treated with VA-ECMO only by using the nearest neighbor method with a caliper of 0.05 and no replacement. After propensity score matching, the balancing of baseline characteristics between both groups was assessed by absolute standard differences (defined as the difference in means, proportions, or ranks divided by the mutual SD), with a value <0.1 considered as not significant.

**Table 1.**
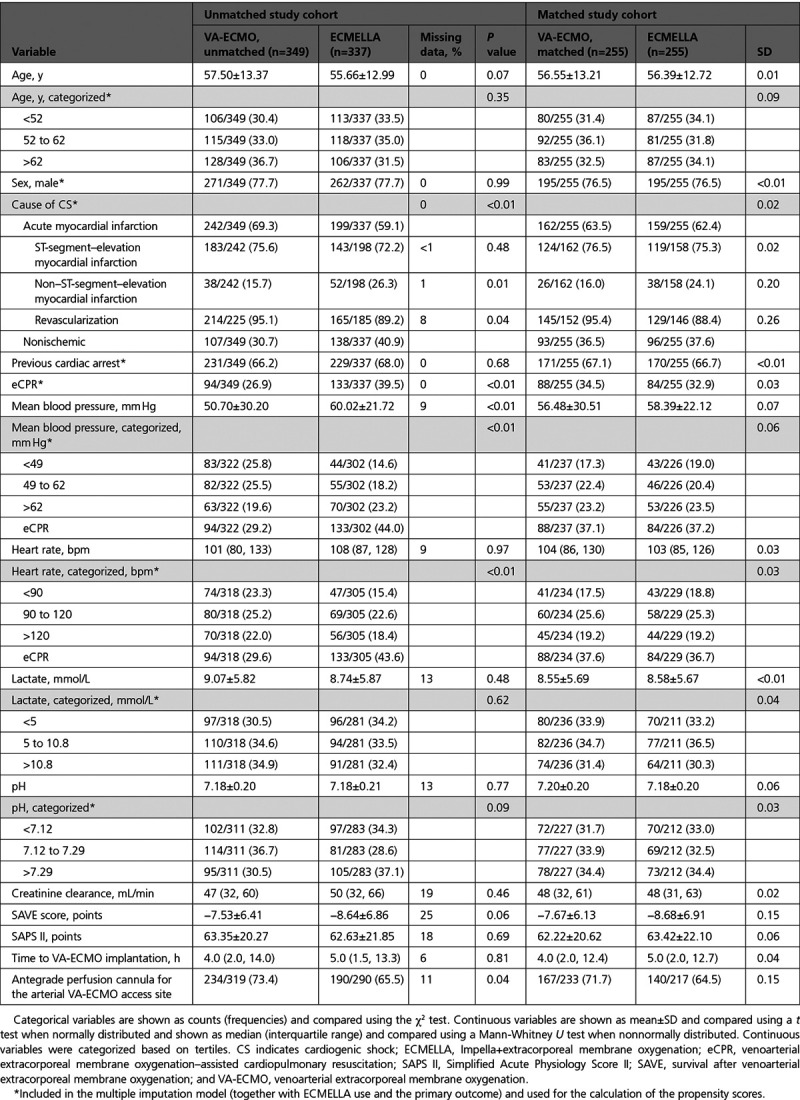
Baseline Characteristics of Unmatched and Matched Study Cohorts

Categorical variables are shown as counts (frequencies) and compared using the χ^2^ test. Continuous variables are shown as mean ± SD and compared using a *t* test when normally distributed and shown as median (interquartile range [IQR]) and compared using a Mann-Whitney *U* test when nonnormally distributed.

The Kaplan-Meier method was used in the unmatched as well as in the matched study cohort to obtain crude 30-day mortality risk and CIs in patients treated with ECMELLA versus VA-ECMO and a Cox regression model was fitted to evaluate the association of ECMELLA use with 30-day mortality. Proportional hazards assumption for ECMELLA use was assessed based on Schoenfeld residuals and met.

To evaluate the association between ECMELLA use and mortality risk in prespecified subgroups of interest, Cox regression models including the interaction between ECMELLA use and the variable representing the subgroup were fitted in the matched study cohort.

To evaluate the association between ECMELLA use and selected safety end points (severe bleeding and intervention because of access site–related ischemia), logistic regression models were fitted in the overall matched study cohort as well as in prespecified subgroups of interest (by including the interaction term between ECMELLA use and the variable representing the subgroup). Logistic regression models were fit to evaluate the association between severe bleeding and access site–related ischemia or ischemic stroke and between use of antegrade perfusion and intervention because of access site–related ischemia and a Cox regression model was fit to evaluate the association between use of antegrade perfusion and 30-day mortality.

To evaluate the association between timing of LV unloading in patients treated with VA-EMO and outcomes, 2 additional matched cohorts were fitted. First, only patients receiving ECMELLA in whom the Impella was implanted before or within 2 hours after the VA-ECMO implantation were considered for matching; eg, matching patients with early LV unloading versus patients treated with VA-ECMO only. Second, only patients receiving ECMELLA in whom the Impella was implanted >2 hours after the VA-ECMO implantation were considered; eg, matching patients with delayed LV unloading versus patients treated with VA-ECMO only. The 2-hour blending period was chosen to reflect time delay in clinical routine; eg, transportation to the catheterization laboratory. In these 2 additional matched cohorts, outcomes were then assessed using the same methods as described above.

All statistical analyses were performed using R 3.5.3.^12^ A *P* value <0.05 was considered statistically significant.

## Results

### Study Cohort

The study cohort is detailed in Table 1. A total of 686 patients with cardiogenic shock were enrolled in the registry and included into the analysis, of whom 349 (51%) were treated with VA-ECMO only (procedures performed between July 2005 and December 2019) and 337 (49%) were treated with ECMELLA (procedures performed between September 2013 and November 2019). In both groups, a femorofemoral access using a percutaneous approach was primarily used for VA-ECMO implantation. After matching, the cohort was restricted to 510 patients with cardiogenic shock: 255 treated with ECMELLA and 255 treated with VA-ECMO.

In the unmatched cohort, mean age was 56.6±13.2 years and 22.3% of the patients were women. Cardiogenic shock was caused by acute myocardial infarction in 64.3% of the patients and 98.3% of these patients were successfully revascularized. A total of 67.1% had previous cardiac arrest. Baseline lactate level was 8.9±5.8 mmol/L and baseline pH level was 7.18±0.21. Although distribution of most baseline characteristics was comparable between groups, nonischemic cardiogenic shock and eCPR were more frequent in patients receiving ECMELLA. In the unmatched ECMELLA group, VA-ECMO was implanted as first device in 149 (44%) patients and Impella was implanted first in 188 (56%) patients. Median interval from Impella to VA-ECMO insertion was 0.0 hours (IQR, −2.0, 3.0), with negative hours indicating VA-ECMO implantation before Impella implantation. An Impella 2.5 was used in 73 (21.7%) cases, an Impella CP in 234 (69.4%) cases, and an Impella 5.0 in 16 (4.7%) cases (data on type of Impella used were missing in 13 [4.2%] cases). Median duration of VA-ECMO use was 5.0 (IQR, 3.0, 8.0) days in the ECMELLA versus 4.0 (IQR, 2.0, 7.0) days in the VA-ECMO only group; median duration of LV unloading in the ECMELLA group was 6.0 (IQR, 2.0, 10.0) days. Of these patients, 18 treated with VA-ECMO (5.4%) and 44 treated with ECMELLA (13.6%) were implanted with a durable LV assist device (*P*<0.01).

After matching, baseline characteristics considered for calculation of the propensity score were well balanced between patients. In the matched ECMELLA group, VA-ECMO was implanted as first device in 111 (44%) patients, and Impella was implanted first in 144 (56%) patients; median interval from implantation of Impella to VA-ECMO was 0.0 (IQR, −2.0, 3.0) hours (negative hours indicate VA-ECMO implantation before Impella). An Impella 2.5 was used in 57 (22.3%) of the cases, an Impella CP in 171 (67.1%) of the cases and an Impella 5.0 in 14 (5.5%) of the cases (missing data on type of Impella used in 14 [5.1%] of the cases). Median duration of VA-ECMO use was 5.0 (IQR, 3.0, 8.0) days in the ECMELLA versus 4.0 (IQR, 2.0, 7.9) days in the VA-ECMO group; median duration of LV unloading in the ECMELLA group was 6.0 (IQR, 2.0, 10.0) days. Of these patients, 16 patients treated with VA-ECMO (6.5%) and 30 patients treated with ECMELLA (12.4%) were implanted with a durable LV assist device (*P*=0.20).

### Outcome Analysis

**Figure 1.**
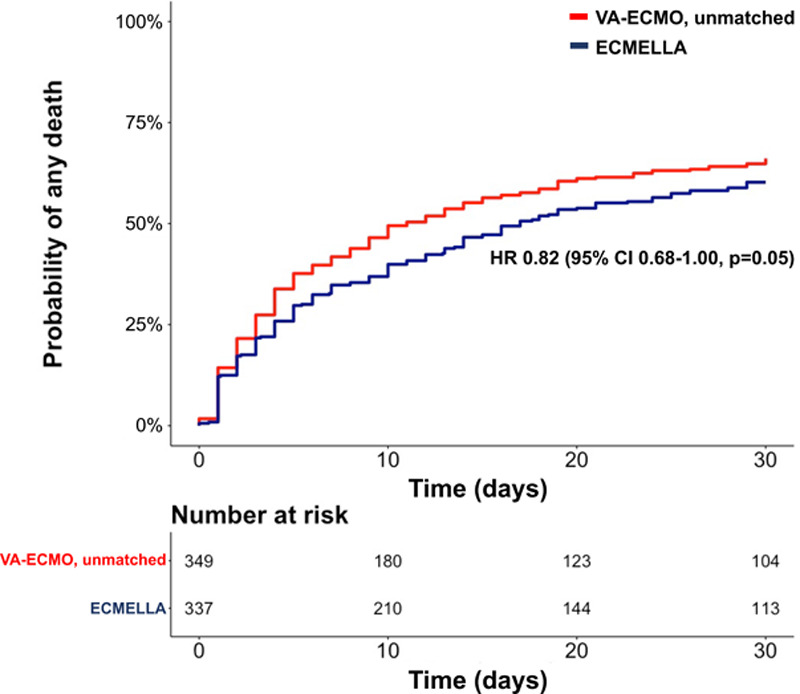
**Kaplan-Meier curve of the unmatched study cohort.** ECMELLA indicates Impella+extracorporeal membrane oxygenation; HR, hazard ratio; and VA-ECMO, venoarterial extracorporeal membrane oxygenation.

In the unmatched cohort, 421 (61.4%) patients died during a median follow-up of 13 (IQR, 3, 30) days (16 [IQR, 4, 30] days in patients treated with ECMELLA versus 10 [IQR, 3, 30] days in patients treated with VA-ECMO). Crude 30-day mortality risk in patients treated with ECMELLA versus VA-ECMO was 60.2% (95% CI, 54.5%–65.3%) versus 66.2% (95% CI, 60.6%–70.9%). Corresponding unadjusted hazard ratio (HR) for ECMELLA use was 0.82 (95% CI, 0.68–1.00; *P*=0.05; Figure 1).

**Figure 2.**
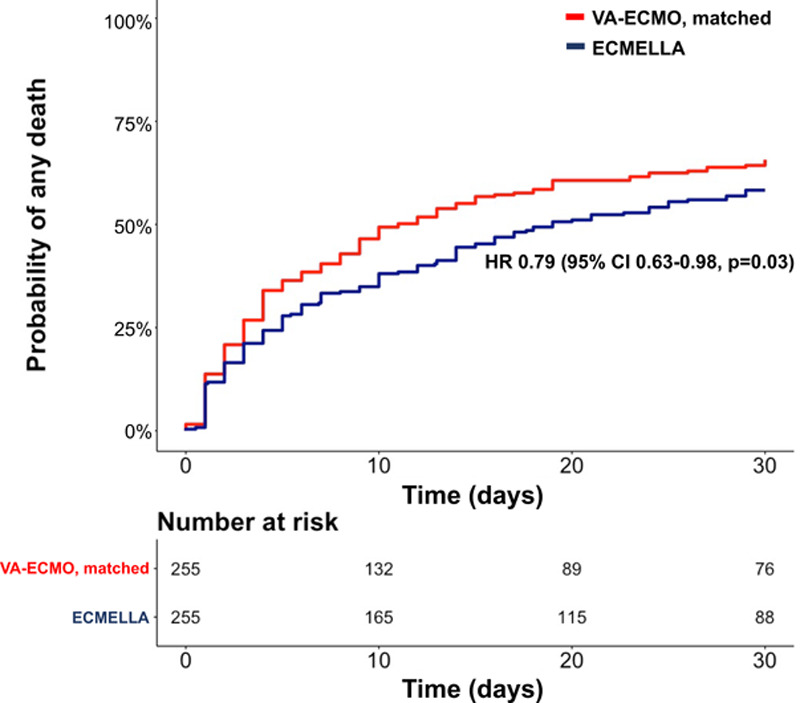
**Kaplan-Meier curve of the matched study cohort.** ECMELLA indicates Impella+extracorporeal membrane oxygenation; HR, hazard ratio; and VA-ECMO, venoarterial extracorporeal membrane oxygenation.

In the matched cohort, 307 (60.2%) patients died during a median follow-up of 14 (IQR, 4, 30) days (17 [IQR, 5, 30] days in patients treated with ECMELLA versus 10 [IQR, 3, 30] days in patients treated with VA-ECMO). Crude 30-day mortality risk in patients treated with ECMELLA versus VA-ECMO was 58.3% (95% CI, 51.6%–64.1%) versus 65.7% (95% CI, 59.2%–71.2%), with an HR for ECMELLA use of 0.79 (95% CI, 0.63–0.98; *P*=0.03; Figure 2).

**Figure 3.**
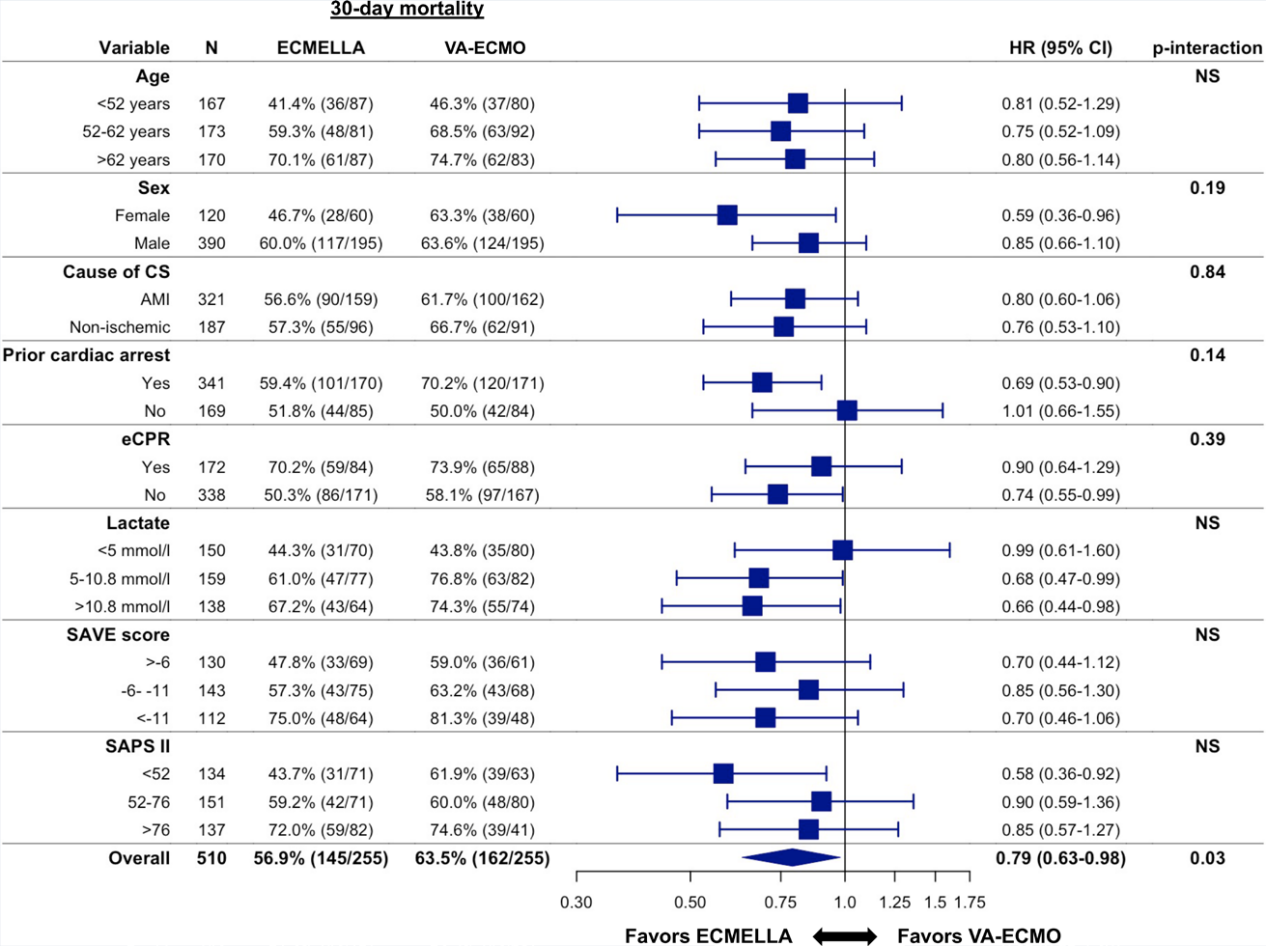
**Association between ECMELLA use and 30-day all-cause mortality in prespecified subgroups**. *P* interaction is 0.79 for age <52 years versus age 52 to 62 years, 0.95 for age <52 years versus age >62 years, and 0.82 for age 52 to 62 years versus age >62 years. *P* interaction is 0.23 for lactate <5 mmol/L versus 5 to 10.8 mmol/L, 0.20 for <5 mmol/L versus >10.8 mmol/L, and 0.90 for 5 to 10.8 mmol/L versus >10.8 mmol/L. *P* interaction is 0.55 for survival after venoarterial extracorporeal membrane oxygenation (SAVE) score >−6 versus −6 to −11, 0.99 for >−6 versus <−11, and 0.52 for −6 to −11 versus <−11. *P* interaction is 0.16 for Simplified Acute Physiology Score II (SAPS II) <52 versus −52 to 76, 0.21 for <52 versus >76, and 0.86 for 52 to 76 versus >76. AMI indicates acute myocardial infarction; CS, cardiogenic shock; ECMELLA, Impella+extracorporeal membrane oxygenation; eCPR, venoarterial extracorporeal membrane oxygenation–assisted cardiopulmonary resuscitation; HR, hazard ratio; NS, nonsignificant; and VA-ECMO, venoarterial extracorporeal membrane oxygenation.

Associations between ECMELLA use and mortality in the prespecified subgroups are reported in Figure 3. There were no significant interactions between ECMELLA use and the variables that defined the subgroups (eg, older versus younger patients, women versus men, patients with versus without cardiogenic shock because of acute myocardial infarction, patients with versus without previous cardiac arrest, patients with versus without eCPR, and patients with higher versus lower lactate level).

### Safety Analysis in the Matched Study Cohort

**Figure 4.**
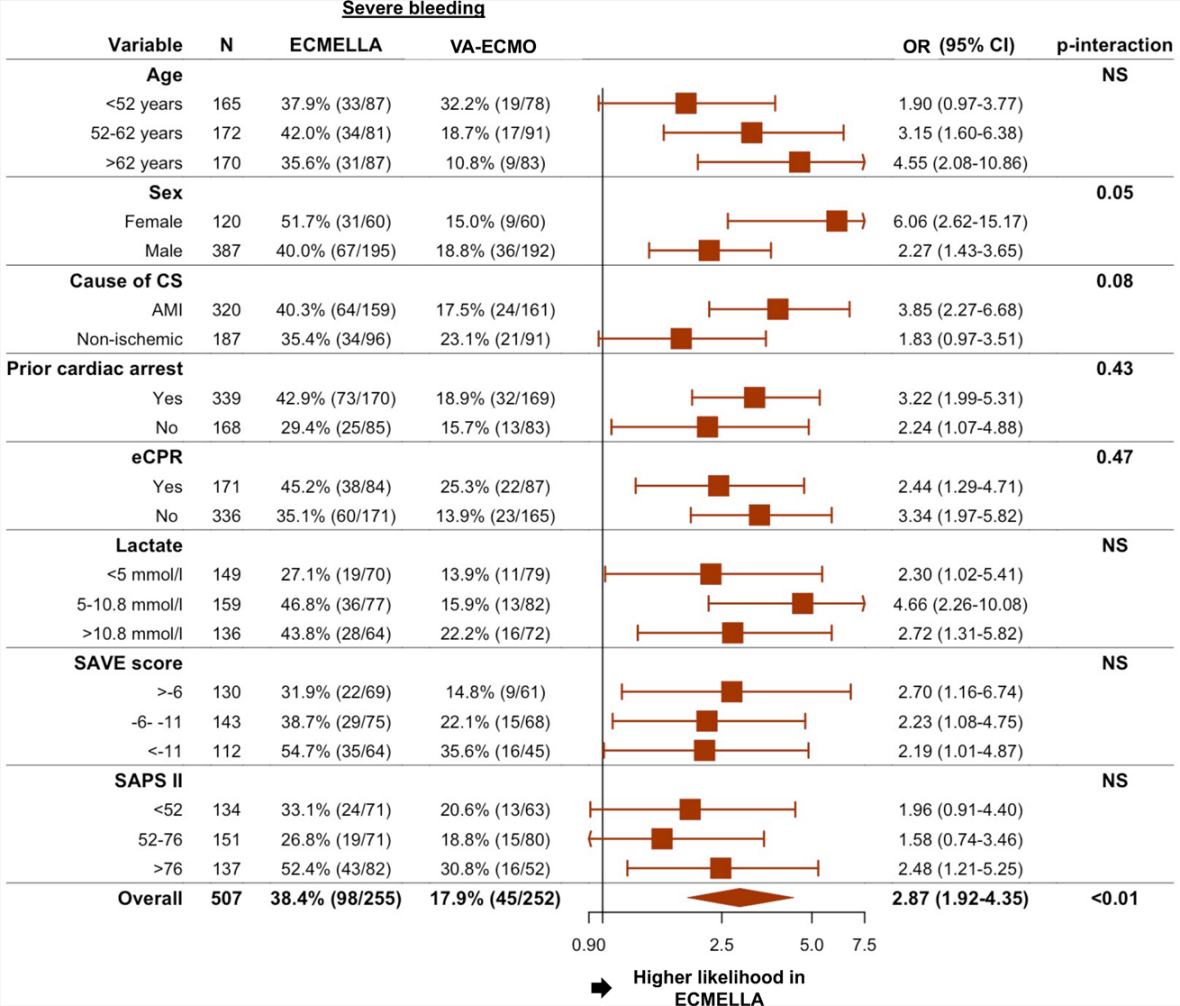
**Association between ECMELLA use and severe bleeding in prespecified subgroups**. *P* interaction is 0.30 for age <52 years versus age 52 to 62 years, 0.11 for age <52 years versus age >62 years, and 0.50 for age 52 to 62 years versus age >62 years. *P* interaction is 0.21 for lactate <5 mmol/L versus 5 to 10.8 mmol/L, 0.77 for <5 mmol/L versus >10.8 mmol/L, and 0.32 for 5 to 10.8 mmol/L versus >10.8 mmol/L. *P* interaction is 0.74 for survival after venoarterial extracorporeal membrane oxygenation (SAVE) score >−6 versus −6 to −11, 0.72 for >−6 versus <−11, and 0.97 for −6 to −11 versus <−11. *P* interaction is 0.70 for Simplified Acute Physiology Score II (SAPS II) <52 versus −52 to 76, 0.67 for <52 versus >76, and 0.41 for 52 to 76 versus >76. AMI indicates acute myocardial infarction; CS, cardiogenic shock; ECMELLA, Impella+extracorporeal membrane oxygenation; eCPR, venoarterial extracorporeal membrane oxygenation–assisted cardiopulmonary resuscitation; OR, odds ratio; NS, nonsignificant; and VA-ECMO, venoarterial extracorporeal membrane oxygenation.

The safety analysis in the matched study cohort is detailed in Table 2. In the matched cohort, bleeding complications and hemolysis occurred more frequently in patients treated with ECMELLA versus patients treated with VA-ECMO; eg, severe bleeding was observed in 38.4% of the patients treated with ECMELLA versus 17.9% in the patients treated with VA-ECMO and hemolysis was observed 33.6% of the patients treated with ECMELLA versus 22.4% of the patients treated with VA-ECMO. The rate of interventions because of bleeding was comparable between the groups. In the logistic regression model, the association between ECMELLA use and higher risk of severe bleeding was consistent in all evaluated subgroups (Figure 4). There was an association between severe bleeding and a higher risk of interventions because of access site–related ischemia (odds ratio, 2.24 [95% CI, 1.38–3.62]; *P*<0.01), but not with ischemic stroke (odds ratio, 1.12 [95% CI, 0.52–2.27]; *P*=0.77).

**Table 2.**
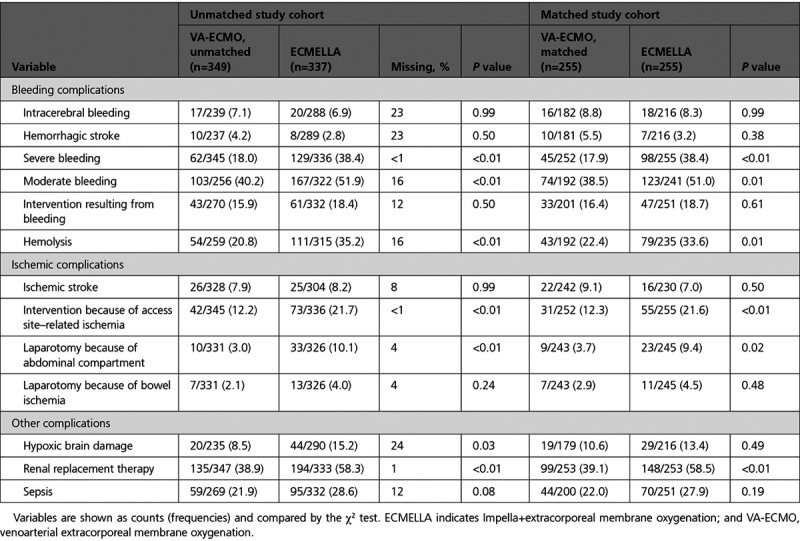
Complications of Unmached and Matched Study Cohorts

**Figure 5.**
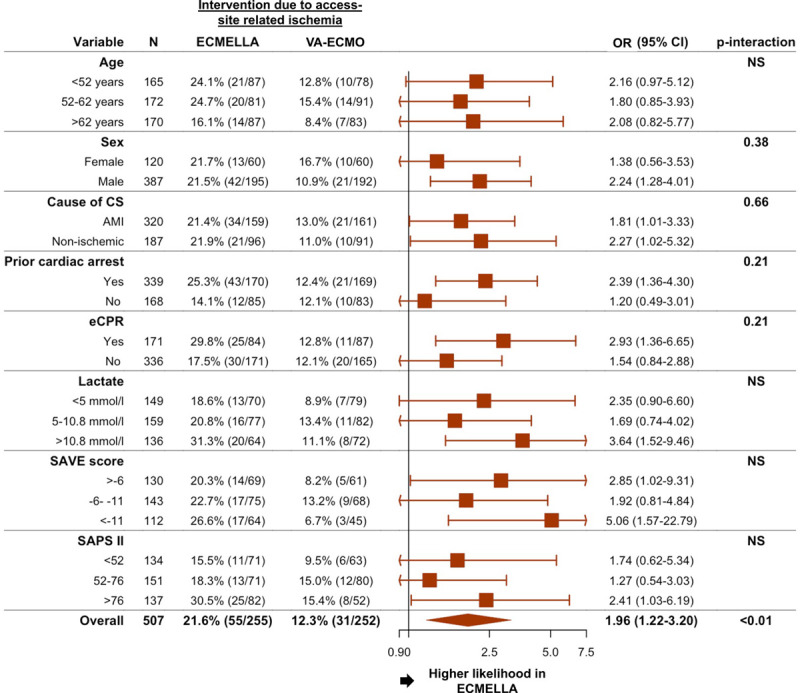
**Association between ECMELLA use and intervention because of access site–related ischemia in prespecified subgroups**. *P* interaction is 0.75 for age <52 years versus age 52 to 62 years, 0.95 for age <52 years versus age >62 years, and 0.82 for age 52 to 62 years versus age >62 years. *P* interaction is 0.62 for lactate <5 mmol/L versus 5 to 10.8 mmol/L, 0.52 for <5 mmol/L versus >10.8 mmol/L, and 0.23 for 5 to 10.8 mmol/L versus >10.8 mmol/L. *P* interaction is 0.58 for survival after venoarterial extracorporeal membrane oxygenation (SAVE) score >−6 versus −6 to −11, 0.51 for >−6 versus <−11, and 0.23 for −6 to −11 versus <−11. *P* interaction is 0.65 for Simplified Acute Physiology Score II (SAPS II) <52 versus −52 to 76, 0.64 for <52 versus >76, and 0.31 for 52 to 76 versus >76. AMI indicates acute myocardial infarction; CS, cardiogenic shock; ECMELLA, Impella+extracorporeal membrane oxygenation; eCPR, venoarterial extracorporeal membrane oxygenation–assisted cardiopulmonary resuscitation; NS, nonsignificant; OR, odds ratio; and VA-ECMO, venoarterial extracorporeal membrane oxygenation.

There was no significant difference in the rate of ischemic strokes or laparotomies because of bowel ischemia between patients treated with ECMELLA versus those treated with VA-ECMO. Interventions because of access site–related ischemia (21.6% of patients treated with ECMELLA versus 12.3% of patients treated with VA-ECMO) and laparotomies because of abdominal compartment syndrome (9.4% of patients treated with ECMELLA and 3.7% of patients treated with VA-ECMO) occurred more frequently in patients treated with ECMELLA. In the logistic regression model, the association between ECMELLA use and a higher likelihood of interventions because of access site–related ischemia was consistent through all evaluated subgroups (Figure 5). Furthermore, there was no significant association between use of antegrade perfusion and 30-day mortality (HR, 0.75 [95% CI, 0.46–1.23]; *P*=0.25) or intervention because of access site–related ischemia (odds ratio, 1.24 [95% CI, 0.73–2.16]; *P*=0.44).

Renal replacement therapy was more frequently used in patients receiving ECMELLA (58.5% versus 39.1%), but there was no difference in the rate of sepsis or hypoxic brain damage.

### Timing of Impella and VA-ECMO Implantation

For this analysis, 222 patients with early LV unloading (eg, Impella implantation before or shortly after VA-ECMO implantation) were matched to 222 patients who were treated with VA-ECMO only and 76 patients with delayed LV unloading (eg, Impella implantation >2 hours after VA-ECMO implantation) were matched to 76 patients who were treated with VA-ECMO only (Tables I through IV in the Data Supplement).

**Figure 6.**
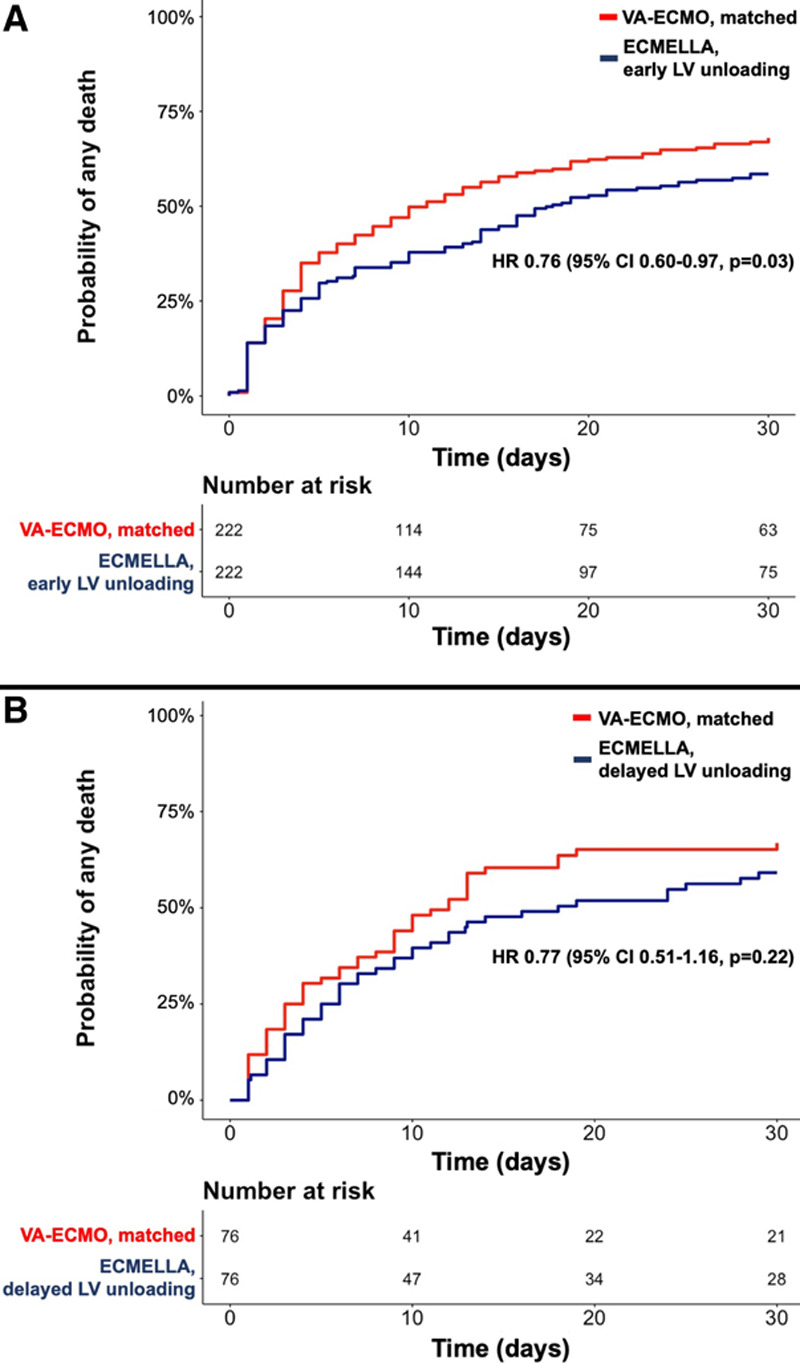
**Kaplan-Meier curves for all-cause mortality in patients receiving ECMELLA treated with early LV unloading and delayed LV unloading versus matched patients treated with only VA-ECMO.** **A**, Only patients receiving ECMELLA in whom the Impella was implanted before or within 2 hours after the VA-ECMO implantation were considered for the matching; eg, matching patients with early LV unloading versus patients treated with VA-ECMO only. B, Only patients receiving ECMELLA in whom the Impella was implanted >2 hours after the VA-ECMO implantation were considered; eg, matching patients with delayed LV unloading versus patients treated with VA-ECMO only. ECMELLA indicates Impella+extracorporeal membrane oxygenation; HR, hazard ratio; LV, left ventricular; and VA-ECMO, venoarterial extracorporeal membrane oxygenation.

In this subanalysis, as compared with VA-ECMO without unloading, early LV unloading was associated with lower 30-day mortality (HR, 0.76 [95% CI, 0.60–0.97]; *P*=0.03; Figure 6), whereas delayed LV unloading was not (HR, 0.77 [95% CI, 0.51–1.16]; *P*=0.22; Figure 6).

A similar trend toward higher incidence of bleeding/ischemic complications with ECMELLA use was seen in both matched cohorts, eg, patients with early LV unloading and patients with delayed LV unloading.

## Discussion

In this large, international, multicenter cohort study of patients with cardiogenic shock, LV unloading with an Impella on top of VA-ECMO was associated with a 21% lower 30-day mortality compared with VA-ECMO alone. The association with a lower mortality risk was consistent through all tested subgroups, including older versus younger patients, female versus male patients, patients with versus without cardiogenic shock because of acute myocardial infarction, patients with versus without previous cardiac arrest, patients with versus without eCPR, as well as patients with higher versus lower lactate level. Complications, including bleeding, hemolysis, and limb ischemia, were more frequently observed in the ECMELLA group.

### Contemporary Use of VA-ECMO in Cardiogenic Shock

VA-ECMO use addresses the central problem of severely reduced cardiac output in cardiogenic shock by providing adequate tissue perfusion.^3^ However, increased afterload because of retrograde aortic perfusion interferes with myocardial recovery and could negatively affect outcome.^13^ This adverse hemodynamic influence of VA-ECMO might partially explain the observation that mortality rates in cardiogenic shock have remained at a high level despite increasing use of VA-ECMO.^2^

This study enrolled patients with severe cardiogenic shock; eg, patients presenting with high lactate level, with low pH, and about a third after eCPR. The observed mortality rate in the overall study cohort was 61%, which is consistent with reports from other studies,^3^ and all patients presented with cardiogenic shock class D or E (Society for Cardiovascular Angiography and Interventions).^14^ LV unloading (in the unmatched study cohort) was more frequently used in eCPR cases and in patients with nonischemic cardiogenic shock. This indicates that physicians prefer this approach in sicker patients, eg, Society for Cardiovascular Angiography and Interventions class E, and as a bailout therapy, because there is no evidence-based treatment for patients with nonischemic cardiogenic shock.^1,3,9,14^

Compared with VA-ECMO, ECMELLA use was associated with lower risk of 30-day mortality in the unmatched as well as in the matched study cohort. This association might be explained by the potentially beneficial effect of LV unloading in patients with cardiogenic shock treated with VA-ECMO. Previous studies have indicated that early LV unloading improves myocardial recovery by reduction in preload, which might be linked to better outcomes.^10,13,15^ Both devices were implanted in quick succession in the ECMELLA group, indicating that early initiation of LV unloading might explain the lower observed mortality. Furthermore, addition of an LV unloading mechanism might improve chances of successful VA-ECMO weaning by providing partial cardiac output support.^16^

The finding of a lower mortality risk with ECMELLA was consistent through all tested subgroups. This included patients with versus without cardiogenic shock because of acute myocardial infarction as well as patients with versus without previous cardiac arrest or eCPR. It is increasingly recognized that various diseases can cause cardiogenic shock and that the majority of cases are not caused by acute myocardial infarction.^17,18^ Because the only evidence-based treatment is early revascularization of the culprit lesion in cardiogenic shock caused by acute myocardial infarction, it is important to identify other treatments that cover the whole spectrum of cardiogenic shock.^19,20^

Aside from LV unloading during VA-ECMO with an Impella, other mechanical circulatory support devices that might provide LV unloading are the intraaortic balloon counterpulsation pump and transseptal left atrial cannulation devices/strategies. Although the intraaortic balloon counterpulsation pump failed to show a benefit in a randomized cardiogenic shock trial as a stand-alone therapy, its passive counterpulsation mechanism could alleviate the increase in LV afterload if combined with VA-ECMO.^21^ A recent meta-analysis of observational studies indicated that LV unloading irrespective of the used device/strategy was associated with lower mortality, but did provide a comparison of various unloading strategies.^6^ Although some aspects might favor Impella over intraaortic balloon counterpulsation pump for LV unloading in patients with cardiogenic shock treated with VA-ECMO (eg, active versus passive mechanism, preload versus afterload reduction), the Impella also requires a larger vascular access, which might mitigate its benefits.^22^ Ultimately, randomized trials are needed to confirm the mortality reduction observed with LV unloading and to determine which unloading strategy is optimal for patients with cardiogenic shock.

### Safety Outcomes Associated With ECMELLA Use

In this study, bleeding complications, ischemic complications, and renal replacement therapy occurred more frequently in the ECMELLA versus the VA-ECMO group and the association between ECMELLA use and bleeding/ischemic complications was consistent in several evaluated subgroups. This contrasts earlier studies, which reported comparable complication rates between patients with cardiogenic shock treated with VA-ECMO or ECMELLA.^7,8^ However, these studies had a small sample size and were most likely underpowered to demonstrate a difference. Several mechanisms support the observation of a higher complication rate with ECMELLA use. First, it has been shown that VA-ECMO use alone is associated with an increase in complications.^23^ The addition of a second device, including the need for a second arterial access, increases the likelihood of bleeding/ischemic complications, especially because ultrasound-guided vascular access, which could reduce such complications, is not always feasible in cardiogenic shock.^24^ Second, previous studies have shown that use of Impella alone is associated with bleeding/ischemic complications, which might be explained by the relatively large vascular access required (12/14 French for the Impella 2.5/CP).^25–27^ The underlying mechanism of the Impella (forcing blood through a small inlet and outlet) causes a high shear stress on blood elements and is associated with increased hemolysis.^28^ Third, higher complication rates, especially higher need for renal replacement therapy, bleeding, and access site–related ischemia, might to a certain degree be explained by survivorship bias. Because patients receiving ECMELLA had better survival and therefore a longer follow-up, they were also more exposed to the risk of complications (because they survived long enough to develop complications).

Several techniques have been suggested to reduce complications in patients on mechanical circulatory support, such as use of antegrade perfusion cannulas and dedicated protocols.^29,30^ Ultimately, the finding of lower mortality but more complications in this study highlights the need for appropriate patient selection to optimize the benefit/risk ratio. Future studies are needed to identify factors that increase or decrease the risk of complications in this setting and could be used to guide decision making in regard to ECMELLA use.

### Timing of Impella and VA-ECMO Implantation

In a subanalysis, early LV unloading followed by VA-ECMO (when the Impella was implanted before or shortly after the VA-ECMO) had a persistent association with a lower mortality risk. However, if the LV unloading was delayed (when the Impella was implanted >2 hours after the VA-ECMO), the association was no longer statistically significant. Although early unloading might prevent severe LV distension, additional research is needed to determine the optimal timing of unloading.

### Limitations

The strengths of this study include the use of a large, international, multicenter cohort of patients with cardiogenic shock with broad data available on several prognostic factors and characteristics. This allowed us to perform propensity score matching based on relevant confounders.

The main limitation of this study is its observational design, so that even after matching residual and unmeasured confounding cannot be ruled out. Data on relevant procedural characteristics, such as cannulation size or anticoagulation strategy, and data on baseline characteristics beyond those reported are missing, which might have affected the results. Similarly, hemodynamic data from right heart catheterization or functional echocardiographic data were not captured in this registry, hence we were not able to investigate differences in intracardiac pressures or LV distension between the groups.^31^ Furthermore, data on additional outcomes of interest such as aortic root thrombosis and North–South syndrome were not available. Although the underlying registry covers several hospitals and nations, generalizability to hospitals without sufficient experience on the use of both devices might be limited. Last, the relatively small sample size of the matched cohort, especially in the cohorts that were matched to evaluate the effect of the timing of LV unloading, might have obscured significant differences in the subgroup analyses.

## Conclusions

In this large, international, multicenter cohort study of patients with cardiogenic shock treated with VA-ECMO, LV unloading with an Impella was associated with lower mortality, but also with more bleeding and ischemic complications, compared with VA-ECMO alone. Although this study supports the use of an Impella for LV unloading in patients with cardiogenic shock treated with VA-ECMO, it also calls for appropriate patient selection and very strict vascular access management to optimize the benefit/risk ratio. This study supports performance of randomized controlled trials evaluating LV unloading in patients with cardiogenic shock supported with VA-ECMO.

## Sources of Funding

This study was funded by the University Heart and Vascular Center Hamburg. Dr Schrage was funded by the German Research Foundation and the Else Kröner-Fresenius-Stiftung.

## Disclosures

Dr Schrage reports receiving a speaker’s fee from AstraZeneca, unrelated to the submitted work. Dr Bernhardt reports personal fees from Abbott, Abiomed, BerlinHeart, Medtronic, and Novartis, unrelated to the submitted work. Dr Bezerra reports personal feels from Abiomed, unrelated to the submitted work. Dr Blankenberg reports grants and personal fees from Abbott Diagnostics, Bayer, SIEMENS, and Thermo Fisher; grants from Singulex; and personal fees from Abbott, Astra Zeneca, Amgen, Medtronic, Pfizer, Roche, Siemens Diagnostics, and Novartis, unrelated to the submitted work. Dr Brunner reports personal fees from Abiomed, unrelated to the submitted work. Dr Kirchhof reports research support for basic, translational, and clinical research projects from European Union, British Heart Foundation, Leducq Foundation, Medical Research Council (United Kingdom), and German Center for Cardiovascular Research; support from several drug and device companies active in atrial fibrillation; and has received honoraria from several such companies in the past, but not in the past 3 years. He is listed as inventor on 2 patents held by the University of Birmingham (Atrial Fibrillation Therapy, WO 2015140571; Markers for Atrial Fibrillation, WO 2016012783), unrelated to the submitted work. Dr Lopes reports a T32 postdoctoral training grant from the National Heart, Lung, and Blood Institute (T32 HL007604), unrelated to the submitted work. Dr Morrow is member of the TIMI (Thrombolysis in Myocardial Infarction) study group, which has received institutional research grant support through Brigham and Women’s Hospital from Abbott, Amgen, Anthos Therapeutics, Aralez, AstraZeneca, Bayer HealthCare Pharmaceuticals, Inc, Daiichi-Sankyo, Eisai, GlaxoSmithKline, Intarcia, Janssen, Merck, Novartis, Pfizer, Poxel, Quark Pharmaceuticals, Roche, Takeda, The Medicines Company, and Zora Biosciences, unrelated to the submitted work. Dr Nordbeck reports personal fees from Abiomed, unrelated to the submitted work. Dr Pappalardo reports personal fees from Abiomed unrelated to the submitted work. Dr Reichenspurner reports personal fees from Medtronic and Abiomed, unrelated to the submitted work. Dr Schulze reports grants and personal fees from Abiomed, unrelated to the submitted work. Dr Schwinger reports receiving a speaker’s fee from Berlin Chemie AG, Pfizer Pharma GmbH, Bristol-Meyers Squibb, Bayer Vital GmbH, Daiichi-Sankyo GmbH, and Novartis Pharma GmbH, outside the submitted work. Dr Sinning reports personal fees from Abbott and Abiomed and grants and personal fees from Boston Scientific, Edwards Lifesciences, and Medtronic, unrelated to the submitted work. Dr Varshney reports a T32 postdoctoral training grant from the National Heart, Lung, and Blood Institute (T32HL007604-35), unrelated to the submitted work. Dr Westermann reports receiving a speaker’s fee from AstraZeneca, Bayer, and Novartis, unrelated to the submitted work. Dr Orban reports receiving personal fees from Abbott Medical, AstraZeneca, Abiomed, Bayer vital, BIOTRONIK, Bristol-Myers Squibb, CytoSorbents, Daiichi Sankyo Deutschland, Edwards Lifesciences Services, Sedana Medical, unrelated to the submitted work.

## Supplemental Materials

Data Supplement Tables I–IV

## Supplementary Material


