# Supplementary material for: Left Ventricular Unloading Is Associated With Lower Mortality in Patients With Cardiogenic Shock Treated With Venoarterial Extracorporeal Membrane Oxygenation: Results From an International, Multicenter Cohort Study
Source: Circulation. 2020 Oct 9;142(22):2095–106. doi: 10.1161/CIRCULATIONAHA.120.048792 (PMC7688081; doi:10.1161/CIRCULATIONAHA.120.048792)
Supplement: Supplementary file 1 [file cir-142-2095-s001.pdf]

SUPPLEMENTAL MATERIAL

Left ventricular unloading is associated with lower mortality in cardiogenic shock patients treated with veno-arterial extracorporeal membrane oxygenation - Results from an international, multicenter cohort study

Schrage et al.

Supplemental Table I. Baseline characteristics of ECMELLA patients treated with early left ventricular unloading vs. matched patients treated with VA-ECMO only.

**Variable**	**VA-ECMO, matched (N=222)**	**ECMELLA, early LV unloading (N=222)**	**SD**
Age, years	57.56 (±13.51)	56.05 (±12.94)	0.11
Age, categorized*			0.05
<52 years	68/222 (30.6%)	72/222 (32.4%)	
52-62 years	74/222 (33.3%)	75/222 (33.8%)	
>62 years	80/222 (36.0%)	75/222 (33.8%)	
Sex, male*	174/222 (78.4%)	172/222 (77.5%)	0.02
Cause of CS*			0.04
Acute myocardial infarction	139/222 (62.6%)	143/222 (64.4%)	
ST-elevation myocardial Infarction	106/139 (76.3%)	106/142 (74.6%)	0.04
Non-ST-elevation myocardial Infarction	26/139 (18.7%)	33/142 (23.2%)	0.11
Revascularization	127/131 (96.9%)	121/135 (89.6%)	0.30
Non-ischemic	83/222 (37.4%)	79/222 (35.6%)	
Prior cardiac arrest*	149/222 (67.1%)	142/222 (64.0%)	0.07
eCPR*	82/222 (36.9%)	75/222 (33.8%)	0.07
Mean blood pressure, mmHg	56.22 (±27.30)	60.54 (±23.09)	0.17
Mean blood pressure, categorized*			0.11
<49 mmHg	33/208 (15.9%)	34/201 (16.9%)	
49-62 mmHg	46/208 (22.1%)	39/201 (19.4%)	
>62 mmHg	47/208 (22.6%)	53/201 (26.4%)	
eCPR	82/208 (39.4%)	75/201 (37.3%)	
Heart rate, bpm	104 (82, 133)	105 (86, 126)	0.03
Heart rate, categorized*			0.06
<90 bpm	34/208 (16.3%)	37/202 (18.3%)	
90-120 bpm	52/208 (25.0%)	52/202 (25.7%)	
>120 bpm	40/208 (19.2%)	38/202 (18.8%)	
eCPR	82/208 (39.4%)	75/202 (37.1%)	
Lactate, mmol/l	8.86 (±5.71)	8.75 (±6.01)	0.02
Lactate, categorized*			0.03
<5 mmol/l	68/203 (33.5%)	62/178 (34.8%)	
5-10.8 mmol/l	66/203 (32.5%)	58/178 (32.6%)	
>10.8 mmol/l	69/203 (34.0%)	58/178 (32.6%)	
pH	7.20 (±0.20)	7.17 (±0.21)	0.13
pH, categorized*			0.09
<7.12	61/199 (30.7%)	64/185 (34.6%)	
7.12-7.29	67/199 (33.7%)	61/185 (33.0%)	
>7.29	71/199 (35.7%)	60/185 (32.4%)	
Creatinine clearance, ml/min	47 (31, 60)	49 (31, 63)	0.17
SAVE score, points	-7.92 (±6.14)	-8.48 (±7.25)	0.08
SAPS II, points	63.23 (±20.37)	61.87 (±22.38)	0.06
Time to VA-ECMO implantation, hours	4.0 (2.0, 12.9)	5.0 (2.0, 16.7)	0.09
Antegrade perfusion cannula for the arterial VA-ECMO access site	144/205 (70.2%)	134/195 (68.7%)	0.03

Categorical variables are shown as counts (frequencies) and compared by the χ^2 test. Continuous variables are shown as mean (±standard deviation) and compared by t-test when normally distributed; and shown as median (interquartile range) and compared by Man-Whitney U test when non-normally distributed. Variables marked with * were included in the multiple imputation model (together with ECMELLA use and the primary outcome) and were used for the calculation of the propensity-scores. Continuous variables were categorized based on tertiles. VA-ECMO: veno-arterial extracorporeal membrane oxygenation therapy; ECMELLA: Impella+VA-ECMO; LV: left ventricular; SD: absolute

standard difference; CS: cardiogenic shock; eCPR: VA-ECMO-assisted cardiopulmonary resuscitation; SAVE score: Survival after veno-arterial ECMO score; SAPS II: Simplified Acute Physiology Score II.

Supplemental Table II. Complications in ECMELLA patients treated with early left ventricular unloading vs. matched patients treated with VA-ECMO only.

	**VA-ECMO, matched (N=222)**	**ECMELLA, early LV unloading (N=222)**	**P**
Bleeding complications			
Intracerebral bleeding	14/160 (8.8%)	12/190 (6.3%)	0.51
Hemorrhagic stroke	7/158 (4.4%)	4/190 (2.1%)	0.35
Severe bleeding	45/219 (20.5%)	89/221 (40.3%)	<0.01
Moderate bleeding	69/172 (40.1%)	102/215 (47.4%)	0.18
Intervention due to bleeding	33/178 (18.5%)	48/219 (21.9%)	0.48
Hemolysis	39/170 (22.9%)	65/206 (31.6%)	0.08
Ischemic complications			
Ischemic stroke	16/207 (7.7%)	12/197 (6.1%)	0.65
Intervention due to access-site related ischemia	24/219 (11.0%)	44/221 (19.9%)	0.01
Laparotomy due to abdominal compartment	8/213 (3.8%)	23/217 (10.6%)	0.01
Laparotomy due to bowl ischemia	6/213 (2.8%)	7/217 (3.2%)	0.99
Other complications			
Hypoxic brain damage	11/156 (7.1%)	29/191 (15.2%)	0.03
Renal replacement therapy	89/221 (40.3%)	134/219 (61.2%)	<0.01
Sepsis	39/177 (22.0%)	58/219 (26.5%)	0.37

Variables are shown as counts (frequencies) and compared by the χ^2 test. VA-ECMO: veno-arterial extracorporeal membrane oxygenation therapy; ECMELLA: Impella+VA-ECMO; LV: left ventricular.

Supplemental Table III. Baseline characteristics of ECMELLA patients treated with delayed left ventricular unloading vs. matched patients treated with VA-ECMO only.

**Variable**	**VA-ECMO, matched (N=76)**	**ECMELLA, delayed LV unloading (N=76)**	**SD**
Age, years	56.04 (±14.01)	55.64 (±13.32)	0.03
Age, categorized*			0.09
<52 years	23/76 (30.3%)	25/76 (32.9%)	
52-62 years	28/76 (36.8%)	29/76 (38.2%)	
>62 years	25/76 (32.9%)	22/76 (28.9%)	
Sex, male*	56/76 (73.7%)	60/76 (78.9%)	0.12
Cause of CS*			0.13
Acute myocardial infarction	47/76 (61.8%)	42/76 (55.3%)	
ST-elevation myocardial Infarction	35/47 (74.5%)	28/42 (66.7%)	0.17
Non-ST-elevation myocardial Infarction	9/47 (19.1%)	14/42 (33.3%)	0.33
Revascularization	42/46 (91.3%)	31/36 (86.1%)	0.17
Non-ischemic	29/76 (38.2%)	34/76 (44.7%)	
Prior cardiac arrest*	54/76 (71.1%)	55/76 (72.4%)	0.03
eCPR*	33/76 (43.4%)	30/76 (39.5%)	0.08
Mean blood pressure, mmHg	56.46 (±31.09)	55.07 (±16.51)	0.06
Mean blood pressure, categorized*			0.23
<49 mmHg	11/73 (15.1%)	10/64 (15.6%)	
49-62 mmHg	13/73 (17.8%)	15/64 (23.4%)	
>62 mmHg	16/73 (21.9%)	9/64 (14.1%)	
eCPR	33/73 (45.2%)	30/64 (46.9%)	
Heart rate, bpm	104 (83, 131)	110 (93, 127)	0.14
Heart rate, categorized*			0.16
<90 bpm	12/72 (16.7%)	8/66 (12.1%)	
90-120 bpm	16/72 (22.2%)	15/66 (22.7%)	
>120 bpm	11/72 (15.3%)	13/66 (19.7%)	
eCPR	33/72 (45.8%)	30/66 (45.5%)	
Lactate, mmol/l	7.51 (±5.61)	8.80 (±5.76)	0.23
Lactate, categorized*			0.26
<5 mmol/l	29/66 (43.9%)	21/67 (31.3%)	
5-10.8 mmol/l	20/66 (30.3%)	25/67 (37.3%)	
>10.8 mmol/l	17/66 (25.8%)	21/67 (31.3%)	
pH	7.24 (±0.21)	7.19 (±0.21)	0.25
pH, categorized*			0.25
<7.12	17/67 (25.4%)	23/63 (36.5%)	
7.12-7.29	18/67 (26.9%)	13/63 (20.6%)	
>7.29	32/67 (47.8%)	27/63 (42.9%)	
Creatinine clearance, ml/min	49 (36, 63)	50 (34, 70)	0.09
SAVE score, points	-6.61 (±4.94)	-8.62 (±6.40)	0.35
SAPS II, points	61.06 (±20.72)	64.01 (±21.43)	0.14
Time to VA-ECMO implantation, hours	4.0 (2.0, 9.9)	3.0 (1.0, 8.3)	0.14
Antegrade perfusion cannula for the arterial VA-ECMO access site	51/73 (69.9%)	38/64 (59.4%)	0.22

Categorical variables are shown as counts (frequencies) and compared by the χ^2 test. Continuous variables are shown as mean (±standard deviation) and compared by t-test when normally distributed; and shown as median (interquartile range) and compared by Man-Whitney U test when non-normally distributed. Variables marked with * were included in the multiple imputation model (together with ECMELLA use and the primary outcome) and were used for the calculation of the propensity-scores. Continuous variables were categorized based on tertiles. VA-ECMO: veno-arterial extracorporeal membrane oxygenation therapy; ECMELLA: Impella+VA-ECMO; LV: left ventricular; SD: absolute

standard difference; CS: cardiogenic shock; eCPR: VA-ECMO-assisted cardiopulmonary resuscitation; SAVE score: Survival after veno-arterial ECMO score; SAPS II: Simplified Acute Physiology Score II.

Supplemental Table IV. Complications in ECMELLA patients treated with early left ventricular unloading vs. matched patients treated with VA-ECMO only.

	**VA-ECMO, matched (N=76)**	**ECMELLA, delayed LV unloading (N=76)**	**P**
Bleeding complications			
Intracerebral bleeding	3/58 (5.2%)	8/65 (12.3%)	0.29
Hemorrhagic stroke	3/57 (5.3%)	4/65 (6.2%)	0.99
Severe bleeding	12/76 (15.8%)	27/76 (35.5%)	<0.01
Moderate bleeding	23/64 (35.9%)	44/68 (64.7%)	<0.01
Intervention due to bleeding	10/64 (15.6%)	8/74 (10.8%)	0.56
Hemolysis	9/59 (15.3%)	24/70 (34.3%)	0.02
Ischemic complications			
Ischemic stroke	4/70 (5.7%)	6/73 (8.2%)	0.80
Intervention due to access-site related ischemia	8/76 (10.5%)	16/76 (21.1%)	0.12
Laparotomy due to abdominal compartment	3/76 (3.9%)	4/70 (5.7%)	0.91
Laparotomy due to bowl ischemia	2/76 (2.6%)	4/70 (5.7%)	0.60
Other complications			
Hypoxic brain damage	5/57 (8.8%)	6/65 (9.2%)	0.99
Renal replacement therapy	34/76 (44.7%)	37/75 (49.3%)	0.69
Sepsis	8/64 (12.5%)	26/74 (35.1%)	<0.01

Variables are shown as counts (frequencies) and compared by the χ^2 test. VA-ECMO: veno-arterial extracorporeal membrane oxygenation therapy; ECMELLA: Impella+VA-ECMO; LV: left ventricular.
